# Pre-Clinical Insights into the Iron and Breast Cancer Hypothesis

**DOI:** 10.3390/biomedicines9111652

**Published:** 2021-11-09

**Authors:** Henry J. Thompson, Elizabeth S. Neil, John N. McGinley

**Affiliations:** Cancer Prevention Laboratory, Colorado State University, Fort Collins, CO 80523, USA; elizabeth.neil@colostate.edu (E.S.N.); john.mcginley@colostate.edu (J.N.M.)

**Keywords:** iron, breast cancer, oxidative damage

## Abstract

Population studies, systematic reviews, and meta-analyses have revealed no relationship between iron status and breast cancer, a weak positive association, or a small protective effect of low iron status. However, in those studies, the authors concluded that further investigation was merited. The set of experiments reported here used preclinical models to assess the likely value of further investigation. The effects of iron status on the initiation and promotion stage of mammary carcinogenesis are reported. Using the classical model of cancer initiation in the mammary gland, 7,12 dimethyl-benz[α]anthracene-induced carcinogenesis was unaffected by iron status. Similarly, excess iron intake showed no effect on the promotion stage of 1-methyl-1-nitrosurea-induced mammary carcinogenesis, though iron deficiency exerted a specific inhibitory effect on the carcinogenic process. Though iron-mediated cellular oxidation is frequently cited as a potential mechanism for effects on breast cancer, no evidence of increased oxidative damage to DNA attributable to excess iron intake was found. The reported preclinical data fail to provide convincing evidence that the further evaluation of the iron–breast cancer risk hypotheses is warranted and underscore the value of redefining the referent group in population-based studies of iron–cancer hypotheses in other tissues.

## 1. Introduction

Iron is an essential dietary trace element. It is required for a broad range of biological functions. Iron deficiency anemia was discovered in 1852 by Karl Vierordt. Over the next century, the details of iron regulation were elucidated, as detailed in [1]. Because of the manner in which iron is metabolized in mammalian species [2], it continues to be regarded as a nutrient of concern in terms of both inadequate intake, which generally manifests as iron deficiency anemia, and excessive accumulation, generally in iron overload diseases that have an inherited component [3,4,5]. However, given the key functions of iron in mammalian systems and concerns about the consequences of inadequate intake, the fortification of foods and use of iron supplements is widespread. Not surprisingly, both within and across major global population centers, the range of iron status is wide. Accordingly, there continues to be an ongoing effort to determine the effects of differences in iron status on health and well-being, as well as disease pathogenesis, e.g., in cancer [6].

Regarding disease pathogenesis and the effects of iron status on breast cancer, which is the focus of the work reported here, a review of the literature revealed that this topic appears to stimulate interest in cycles of roughly 10 years. A previous burst in published activity occurred in the timeframe of 2008–2013 [7,8,9,10,11,12,13,14,15,16,17,18,19]. The prevailing issues underlying those investigations included the impact of iron deficiency in limiting processes related to tumor development and the effects of excess iron-induced oxidative stress on the carcinogenic process. Papers published between 2017 and 2021 [6,20,21,22,23,24,25,26,27,28,29,30,31,32,33,34,35,36,37,38,39,40,41,42,43,44,45] indicated that this cycle of interest is once again repeating. The prevailing hypotheses include not only those that were prominent in the 2008–2013 timeframe but also those that are centered on iron metabolism as regulated by hepcidin, with extension to how the gut microbiome and immune system might be involved in effect mediation. There has also been a clear shift toward issues germane to cancer treatment. 

Given the results recently reported from large-population cohorts and the mechanisms being discussed [26,32,34,40,42,46], the work reported here was intended to take advantage of the value of preclinical studies in the deconstruction of complex population observations. Three questions highly relevant to understanding the population data that are being reported are addressed: (1) are the limited and conflicting effects of iron status on breast cancer risk observed in population studies consistent with the impact of iron status observed in preclinical studies of mammary cancer initiation or promotion? (2) are the protective effects of low iron status observed in population studies potentially attributable to iron status per se? and (3) do systemic biomarkers of iron status reflect iron status in the mammary gland and are tissue iron levels positively associated with oxidative cellular damage? The implications of these findings in the pursuit of iron–cancer hypotheses in other organ sites are also discussed.

## 2. Materials and Methods

### 2.1. Animal Experiments

Female Sprague Dawley rats were obtained from Taconic Farms (Germantown, NY, USA) at 21 days of age, housed three per cage, and maintained in an environmentally controlled room at 22 °C with 50% relative humidity and a 12 h light/12 h dark cycle. Rats were fed a purified diet formulation that only varied in the amount of iron that was provided [47]. Details of the design of each experiment are provided with the presentation of results. The methods for the detection and classification of mammary tumors have been previously described [48,49].

### 2.2. Assays and Chemical Analysis

Using the tissue that was quick-frozen in liquid nitrogen at necropsy, pieces of liver and mammary gland were processed for routine tissue iron analysis [50]. Weighed amounts of tissue were wet-ashed in concentrated nitric and perchloric acid (3:1, *v*/*v*), and tissue digests were diluted to volume. Iron was assayed by atomic absorption spectroscopy (Perkin Elmer, Model 405 spectrophotometer) using an air–acetylene flame and standard procedures of operation, as described by the manufacturer (Perkin Elmer, Norwalk, CT, USA). Liver microsomes were isolated, and their lipid extract was assayed for thiobarbituric acid reactive material, an indicator of lipid peroxidation [51].

Ferritin concentration in rat serum was estimated using a BIO-RAD Quantimmune^®^ two-site immunoradiometric assay, catalog no. 190-2001 (BIO-RAD, Hercules, California, USA). The assay kit is designed for measuring human ferritin and was modified for use with rat serum. 8-OHdG and dG were quantitated by use of reverse phase HPLC that utilized electrochemical and spectrophotometric detection. The method we employed was based on that of Floyd et al. [52,53], with some modifications. The separation was isocratically performed on a 4.63250 mm Rainin Microsorb C18 column (5 µm and 100 A) with a mobile phase of 8.2% methanol in 50 mM potassium phosphate buffer, pH 5.5, delivered at 1 mL/min. The detection of 8-OHdG was achieved with an ESA Coulochem Model 5100A electrochemical detector equipped with a model 5011 analytical cell (Dionex, Sunnyvale, CA, USA). Detector potentials were set as follows: guard cell of 10.43 V, detector one of 10.12 V, and detector two of 10.38 V. 8-OHdG was measured as current at detector two. dG was monitored by absorbance at 290 nm with a Shimadzu SPD-10AV spectrophotometric detector (Shimadzu Scientific Instruments, Columbia, MD, USA) installed downstream from the electrochemical detector. Results are reported as moles 8-OHdG per 106 moles dG. 8-OHdG was generously provided by R.A. Floyd; dG was purchased from Boeringer Mannheim (Mannheim, Germany).

### 2.3. Statistical Analysis

Biochemical data were first subjected to exploratory analyses to confirm that the assumptions of normality were met. They were then subjected to analyses of variance and/or regression analyses, as recommended by Snedecor and Cochran [54]. The tumor count data had a Poisson distribution. Therefore, non-parametric methods of analysis recommended by Peto [55] were used to evaluate differences among groups in the final incidence of cancer and tumor multiplicity. Analyses were performed using the Systat statistical analysis system, version 13.0 (Systat Software, Inc., San Jose, CA, USA).

## 3. Results and Discussion

Recently reported results from population studies, systematic reviews, and meta-analyses revealed no relationship between iron status and breast cancer, a weak positive association, or a small protective effect of low iron status [6,20,21,22,23,24,25,26,27,28,29,30,31,32,33,34,35,36,37,38,39,40,41,42,43,44,45]. However, in most of those studies, the authors concluded that further investigation was merited. The set of experiments reported here used preclinical models to assess the likely value of further investigation. The usefulness of an animal model for deconstructing population-based observations depends on the questions being addressed and the appropriateness of the animal model relative to those questions. The rat has been widely used in the investigation of iron nutrition in humans [56] and was the species used in these experiments since it is also recognized that chemically induced mammary carcinogenesis in the rat has comparable histogenesis and pathogenesis to the breast cancers that occur in women [57]. Moreover, in population studies, it is generally not feasible to distinguish the effects of iron status on the initiation vs promotion stage of carcinogenesis. Initiation is defined as the process during which heritable modifications of DNA with carcinogenic potential are induced. Promotion is the process during which cancer-initiated cells clonally develop due to a selective growth advantage relative to adjacent non-initiated epithelial cells, a process terminating in the establishment of observable pathologies that can be histopathologically classified. The effects of iron status on the initiation and promotion stage of mammary carcinogenesis are reported here.

### 3.1. Effect of Iron Status on the Initiation Stage of Mammary Carcinogenesis

For the purposes of this investigation, the initiation of the carcinogenic process was limited to studying the impact of iron status on polycyclic aromatic hydrocarbon (PAH)-induced mammary cancer. The classic model of initiation in the mammary gland utilizes 7,12 dimethylbenzanthracene (DMBA) [58]. This PAH is metabolized to the proximate carcinogen, DMBA-3,4-diol-1,2-epoxide, by both liver and mammary tissue via the P-450 cyclo-oxygenase system (CYP). Since iron is required for the catalytic activity of some CYP proteins, it is possible that differences in iron status could affect carcinogen metabolism and the subsequent development of cancer.

Based on previous work [47,59] and the established requirements of the rat for dietary iron, an initial experiment was conducted to determine iron status in the liver and mammary gland on the day carcinogen was injected into the rat. Weanling rats were fed 6, 35, or 350 ppm of dietary iron. These levels of dietary iron resulted in a gradient of tissue iron levels in the liver and mammary gland without affecting the rate of growth (Table 1).

Based on those data, an experiment was designed to test the effect of iron status on DMBA-initiated mammary carcinogenesis. Briefly, 21-day-old rats were randomized into one of three dietary groups and maintained on their respective diets (6, 35, or 350 ppm of dietary iron provided as ferrous fumarate) until 64 days of age, i.e., 14 days post carcinogen, with carcinogen being administered at 50 days of age. DMBA (10 mg/rat) was administered by gavage. Operationally, 64 days of age is considered the end of the initiation stage of the carcinogenic process. At that time, all rats were switched to the 35 ppm of iron diet for the remainder of the study, which was terminated 90 days post carcinogen administration. Across this gradient of iron status, the initiation phase of mammary carcinogenesis induced by DMBA was unaffected (Table 2).

It is well known that both benign and malignant pathologies are induced in the mammary gland by DMBA. Here, iron status had no effect on the types of pathologies that were observed (Table 3).

From a clinical perspective, this experiment was designed under consideration that the standard of care is to treat iron deficiency anemia when it is detected. Therefore, the experiment described above modeled low but not deficient iron status, as well as adequate iron status and a level of iron stores that would occur when a dietary iron supplement is taken habitually. Under these circumstances, which are consistent with work reported in population studies of the iron–breast cancer hypothesis, we obtained no evidence that iron status affects CYP-mediated carcinogen metabolism, as detected by differences in tumor induction. 

### 3.2. Effect of Iron Status during the Promotion Phase of Mammary Carcinogenesis

The operational definition of the promotion phase of chemically induced mammary carcinogenesis is that it begins after carcinogen metabolites are no longer detected and the repair of carcinogen-induced damage has ceased. In inducing mammary cancer using MNU, animals were randomized to experimental groups 7 days post carcinogen injection.

**Figure 1:**
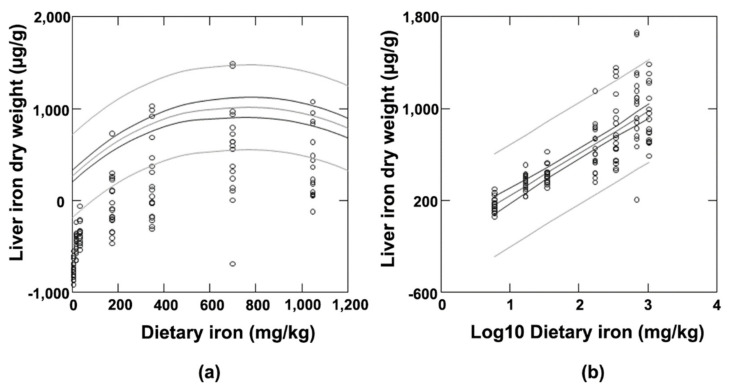
Iron regression analysis. (**a**) Polynomial regression analysis, r2 = 0.062, *p* < 0.001. For the linear component, *p* < 0.001; for the quadratic component, *p* = 0.001; for the cubic component, *p* = 0.047. (**b**) Linear regression, r2 = 0.062, *p* < 0.0001.

In an initial experiment using non-carcinogen-treated rats, a seven-level iron dose response study was conducted. The approach to quantifying body iron stores was based on measuring liver iron, recognizing that the liver is a primary site of iron storage in both humans and rats (Table 4 and Figure 1). As expected, body iron stores, as reflected in hepatic iron concentration, increased with increasing dietary iron (Table 4). The polynomial regression of the non-transformed dose data indicated that the increase was curvilinear (Figure 1A). When the dose data were log-transformed, the increase was log linear (Figure 1B). These findings are consistent with the robust control of iron absorption in the intestine. There was a clear clustering in response of the lowest three and highest four levels of dietary iron, an observation used in the analysis of the carcinogenesis data (Figure 1B).

Using the same dietary concentrations of iron described in Figure 1, a mammary carcinogenesis experiment was initiated using 50 rats per group in order to detect smaller effect sizes among dietary iron concentrations given that the population data indicated that small differences would likely be observed (Table 5). Consistent with other preclinical reports, effects were modest and not statistically significant [47]. More importantly, the data were consistent with three population studies published between 2019 and 2021 [6,20,21,22,23,24,25,26,27,28,29,30,31,32,33,34,35,36,37,38,39,40,41,42,43,44,45]. Specifically, we evaluated the data (Table 5) in a manner that paralleled the common approach in epidemiology, contrasting low versus high iron status, i.e., by comparing groups 1–3 vs. 4–7: the cancer incidence was 63.3 vs. 70.0 (RR = 1.10 (0.95, 1.20; *p* = 0.098)). This relative risk is strikingly similar to the values reported in [31,40]. We also used these data to consider the potential impact of low iron status. When the lowest level of dietary iron was contrasted to the response in all other groups, the relative risk for breast cancer was 0.88 (0.69, 1.12), *p* = 0.143. This finding is similar to the relative risk observed in [23], where low iron status was reported to be protective, and is consistent with an earlier preclinical report [59].

### 3.3. Effect of Deficient Iron Status on the Promotion Phase of Mammary Carcinogenesis

In previous published work [59], it was shown that 3 ppm of dietary iron hindered weaning-induced symptoms of iron deficiency and inhibited the promotion phase of mammary carcinogenesis. However, the inhibition of the carcinogenic process was reversed when rats were given an adequate concentration of dietary iron. Since low iron status may be the driver of clinical reports of the iron-associated enhancement of breast cancer risk, given the reference group in population studies is usually comprised of individuals with the lowest iron status, we decided that it was important to investigate the impact of deficient dietary iron status on mammary carcinogenesis using the gold standard experimental design, i.e., paired feeding. This was deemed necessary since iron deficiency suppresses body weight gain, which itself also inhibits mammary carcinogenesis [60].

In this experiment, individually housed female Sprague Dawley rats were injected with MNU (37.5 mg/kg of body weight at 21 days of age (day of weaning)) and all rats ate an iron-deficient diet for the next 7 days. Thereafter, rats were randomized to continue eating the iron-deficient diet ad libitum, an iron-adequate diet ad libitum, or the iron-adequate diet in the amount consumed by the iron-deficient group. The carcinogenesis protocol is referred to as the rapid emergence model for breast cancer, as published by us [61]. Using this model, the experimental duration was 45 days post carcinogen. All observable mammary pathologies (at 5× magnification) were excised at necropsy and histopathologically classified. Only pathologies that were mammary carcinoma are reported. The incidence of mammary cancer and the average number of cancers per rat were significantly reduced in the iron-deficient group in comparison to either its pair-fed control or the ad libitum fed control group (Table 6). Thus, there appeared to be an iron-deficiency-specific effect on the development of mammary cancer. We judge that this is important for population studies since previous cycles of publications suggested that future studies of iron–cancer hypotheses are likely to be reported, if not in breast then minimally in other organ sites [62]. The more meaningful effects of either low or high iron status on cancer risk should be assessed by making the referent group chosen for comparison be the iron-adequate group, as judged using clinical standards of iron status assessment. If this were done, we suspect that the focus of future work would shift from iron overload to the risk/benefit assessment of iron at the intersection of the deficient versus low normal iron boundary. This has merit since concern has been expressed regarding the impact of the unneeded use of iron supplements on the ecology of the gut microbiome [63,64]. 

### 3.4. Examining Other Key Tenants of the Iron Stores Cancer Hypothesis

There are several validated biomarkers of body iron status, and the contemporary literature has generally reported one or more of these biomarkers in assessing the iron status–breast cancer risk hypothesis. However, limited consideration has been given in those studies regarding how indicators of whole body or circulating iron status are correlated with levels of iron or the processes it mediates in the mammary gland or mammary tumors. To our knowledge, there has been scant consideration of this issue in the >1600 literature citations retrieved in PubMed in response to the query “iron and breast cancer”. The following experiments address the knowledge gap.

### 3.5. Effect of Dietary Iron on Oxidative Indices: Diets Fed from 21 to 90 Days of Age

A typical duration of a nutritional feeding study is 28 days; however, our experimental duration was extended to 69 days. The focus was the effects of iron status on cellular oxidation in the liver. Three different planes of iron nutrition were established, as indicated by plasma ferritin concentration—a commonly used biomarker of iron status used in population studies (Table 7). The highest exposure to iron was associated with an increased concentration of oxidative damage to lipids (measured as malondialdehyde reactive material) and to DNA (measured as 8-oxo-dG).

These data indicate that in the liver, a storage organ for iron, oxidative cellular damage measured as lipid peroxidation or DNA oxidation is increased in proportion to the increase in plasma ferritin. However, the modest trend toward an increase in urinary 8-isoprostane F-2 alpha (a useful index of overall in vivo lipid peroxidation) with increasing dietary iron is consistent with the possibility that in vivo, iron may mediate oxidative events in only a limited number of organ sites. It is of interest that iron-associated liver carcinogenesis is an active area of investigation [65,66].

However, for the purposes of this investigation, a primary focus was the mammary gland. No effect of iron status was observed on DNA oxidation in either the mammary gland or mammary tumors (Table 8). These data fail to support a direct relationship between iron status in the mammary gland or mammary tumors and levels of 8-OHdG.

### 3.6. Summary and Implications

Iron is an essential trace nutrient and a potential “chemical toxin” if free iron becomes available within a cell, as explained by the well-characterized Haber–Weiss reaction. However, mammals have an extremely refined system of checks and balances that maximize an organism’s ability to not only assimilate iron when it is needed but also bind iron within the host so that free iron is generally limited, thus minimizing iron-induced oxidation events within the cell. Nonetheless, there have been over 1600 publications on the topic of iron and breast cancer. The magnitude of interest in this topic is undoubtedly due not only to the fact that breast cancer is the leading cause of cancer deaths among women but also because women have a disproportionately large risk for iron deficiency anemia and are therefore arguably the most likely segment of the population to consume iron-fortified foods and to ingest nutritional supplements containing iron. However, population studies and the supporting preclinical studies reported here have demonstrated limited evidence of failed iron homeostasis resulting in increased breast cancer risk with increasing iron status. Rather, the above-reported preclinical studies have made the case that low iron status is protective against cancer and that excess iron intake has limited impact on breast cancer risk. We argue that given the clinical significance of iron deficiency anemia and that the standard of care in response to detection of a deficiency is iron supplementation, the plausibility of exploiting low iron status for breast cancer prevention is essentially nonexistent. Relative to other tissues, perhaps the best case can be made for the investigation of liver cancer risk in cases of iron overload, particularly for individuals genetically predisposed to iron storage disorders. The other area that merits investigation is the impact of iron intake (food, fortified foods, and iron supplements) on intestinal dysbiosis, which has been a matter of concern for over five decades. Given the explosion of interest and technical capability in microbiome research, it is time for definitive investigations of this topic. Nonetheless, despite these statements, the preclinical data reported here—in combination with the results of recent population studies, systematic reviews, and meta-analyses—provide convincing evidence that further evaluations of iron–breast cancer risk hypotheses are not warranted. Whether the intersection of precision nutrition with precision oncology will identify target populations in which iron status may affect breast cancer treatment remains to be determined.

## Figures and Tables

**Table 1:** Effect of dietary iron on host iron status.

Dietary Iron (ppm)	Hemoglobin (g/dL)	Hematocrit (%)	Plasma Ferritin (ng/mL)	Liver Iron (µg/g)	Mammary Gland Iron Fat Free (µg/g)
6	13.1 ± 0.5	40.4 ± 0.8	25.7 ± 10.6 ^a^	143 ± 25 ^a^	96 ± 12 ^a^
35	14.6 ± 0.1	43.9 ± 1.2	89.6 ± 18.2 ^b^	591 ± 51 ^b^	131 ± 25 ^b^
350	14.6 ± 0.1	41.5 ± 2.5	204.5 ± 42.4 ^c^	1006 ± 89 ^c^	148 ± 3 ^b^

N = 10/diet group, means ± SD; values in a column with different superscripts statistically different via ANOVA with post hoc comparisons. If a column has no superscript, differences are not significant.

**Table 2:** Effect of dietary iron on the carcinogenic response to DMBA in the mammary gland.

Dietary Iron (ppm)	Body Weight at Time of DMBA Administration (g)	Body Weight at End of Study (g)	Final Incidence of Mammary Carcinoma (%)	Final Average Number of Carcinomas per Rat
6	171 ± 2	282 ± 4	86.7	4.1
35	173 ± 4	288 ± 10	89.7	3.6
350	172 ± 2	286 ± 6	90.0	4.0

N = 24 rats per group; values are means ± SD.

**Table 3:** Effect of dietary iron on the distribution of tumor types in the mammary gland.

		Dietary Iron at Time of Carcinogen Treatment		
Diagnosis	Comments	Low Fe 6 ppm	Adequate Fe 35 ppm	High Fe 350 ppm
Carcinoma	Comedo	3	3	2
	Cribriform	3	7	1
	Highly vascular	3	4	7
	Invading muscle	1	6	7
	Mucinous			1
	Papillary	8	2	11
	PDCISC	8	4	4
	with FA	7	11	11
	Mixed	91	63	70
	Total	124	100	121
Ductal carcinoma in situ	Comedo	1		
Adenoma		1	2	2
Fibroadenoma		4	7	2

N = 24 rats/diet group; FA, fibroadenoma; Mixed, phenotype consisting of both cribriform and papillary components; PDCISC, predominant ductal carcinoma in situ component.

**Table 4:** Effect of dietary iron on body iron stores measured as hepatic iron concentration.

Dietary Iron (mg/kg)	Liver Iron ^a^ Dry wt (µg/g)
6	159 ± 68
17.5	356 ± 76
35	421 ± 80
175	641 ± 192
350	796 ± 264
700	1028 ± 344
1050	916 ± 240

^a^ N = 16/group; values are means ± SD. Iron provided as ferrous fumarate.

**Table 5:** Effect of dietary iron on the promotion phase of mammary carcinogenesis.

Group	Dietary Iron (mg/kg)	Cancer Incidence ^a^ (%)	Cancer Multiplicity (Number/Rat) ^c^	Body Weights ^b^ (g)
1	6	58.0 (30)	0.86	339 ± 5
2	17.5	70.0 (35)	1.58	342 ± 5
3	35	58.0 (30)	1.04	353 ± 6
4	175	66.0 (33)	1.44	348 ± 5
5	350	80 (40)	1.49	338 ± 6
6	700	62.0 (31)	1.40	332 ± 7
7	1050	72.0 (36)	1.44	331 ± 5

^a^ Group 1–3 vs. 4–7: RR = 1.10 (0.95, 1.20, *p* = 0.098); Group 1 vs all others: RR = 0.88 (0.69, 1.12, *p* = 0.143). ^b^ Values are means ± SD. ^c^ Average number of mammary carcinomas per rat.

**Table 6:** Effect of iron deficiency on the promotion stage of mammary carcinogenesis.

Group	Final AC Incidence (%)	Final AC Multiplicity (Number/Rat) ^c^	Final Body Weights (g)	Body Length (cm)
35 ppm of Fe Ad Lib (N = 24)	87.5 ^a^	3.04 ^a^	203 ± 3 ^a^	37.9 ± 0.2 ^a^
35 ppm of Fe Pair-Fed 3 ppm of Fe (N = 24)	95.8 ^a^	3.13 ^a^	189 ± 3 ^b^	36.2 ± 0.22 ^b^
3 ppm of Fe Ad Lib (N = 24)	54.2 ^b^	1.13 ^b^	177 ± 3 ^b^	36.0 ± 0.2 ^b^

Adenocarcinoma (AC). Values are means ± SD. Values in a column with different superscripts are statistically different, *p* < 0.05. ^c^ Average number of mammary carcinomas per rat.

**Table 7:** Effect of dietary iron on iron status and oxidative stress markers.

Dietary Iron (ppm)	Final Body Weight (g)	Plasma Ferritin (ng/mL)	Liver MDA (pmol/mg) Protein ^a^	Liver 8-OHdG (Residue/10^6^ dG) ^a^	Urinary Isoprostane F-2 Alpha (ng/mg) Creatinine
6	242 ± 7	25.7 ± 10.6	426 ± 29	6.6 ± 0.7	7.2 ± 0.6
35	248 ± 6	89.6 ± 18.2	529 ± 23	7.8 ± 1.3	7.7 ± 0.5
350	248 ± 7	204.5 ± 42.4	665 ± 46	11.3 ± 2.0	8.1 ± 0.5

Values are means ± SD; ^a^ Linear increase, *p* < 0.05.

**Table 8:** Effect of dietary iron on mammary gland and tumor levels of iron and 8-OHdG.

Dietary Iron (ppm)	Mammary Gland Iron (ppm)	Mammary Gland 8-OHdG (Residues/10^6^ dG)	Mammary Tumor Iron (ppm)	Mammary Tumor 8-OHdG (Residues/10^6^ dG)
6	48 ± 3	6.3 ± 0.4	63 ± 22	5.5 ± 0.4
35	71 ± 8	6.2 ± 0.3	75 ± 26	6.6 ± 0.6
350	83 ± 7	7.0 ± 0.5	113 ± 21	6.0 ± 0.3

Values are means ± SD.

## Data Availability

The data presented in this study are available upon request from the corresponding author.
